# Successful management of Klippel–Trenaunay syndrome in a pregnant Asian woman: A case report

**DOI:** 10.1097/MD.0000000000019932

**Published:** 2020-05-08

**Authors:** Li Xiao, Bing Peng, Haibo Qu, Xiaohui Dai, Jinfeng Xu

**Affiliations:** a Department of Obstetrics and Gynecology, West China Second University Hospital of Sichuan University; b Key Laboratory of Birth Defects and Related Diseases of Women and Children (Sichuan University), Ministry of Education; c Department of Medical Imaging, West China Second University Hospital of Sichuan University; d Department of Ultrasonic Medicine, West China Second University Hospital of Sichuan University; e West China School of Medicine, Sichuan University, Chengdu, Sichuan, PR China.

**Keywords:** anemia, hemangioma, KTS, pregnancy

## Abstract

**Rationale:**

Klippel–Trenaunay Syndrome (KTS) is a congenital vascular disease characterized by cutaneous hemangiomas, venous varicosities, and limb hypertrophy. Although extremely rare in pregnant women, the present vascular alterations may be aggravated, consequent to postural and hormonal changes inherent to the pregnancy. Pregnancy is not advised in KTS women due to increased obstetrical risk.

**Patient concerns:**

A 31-year-old pregnancy woman presented with prominent vascularity in pelvis, right lower limb, spleen, and liver at 28 weeks of gestation. We started administration of anticoagulant therapy and obstetrics management.

**Diagnosis:**

MRI and ultrasound revealed that multiple varicosities in her pelvis, right lower limb, spleen, and liver. She was diagnosed with KTS.

**Interventions:**

At her first visit at 28 weeks of gestation, multidisciplinary evaluation had been done. Blood transfusion and iron supplement had been given for anemia correction. Anticoagulant therapy was performed to prevent potential thrombus risk. She had a vaginal delivery with a healthy newborn in her second visit without any complications at the gestation of 36^+6^ weeks due to rupture of preterm membranes.

**Outcomes:**

After successful management, the patient was discharged without any complications 2 days after vaginal delivery. No symptoms of hemorrhage or thrombus were observed. At 6 months follow-up, her right lower toes enlarged obviously, MRI revealed that no obvious changes of hemangiomas was found compared to those during the pregnancy and ultrasound revealed that there was no thrombus in her right lower limb.

**Lessons:**

Patients with KTS can be pregnant and have healthy babies safely with regularly monitor and reasonable treatment during pregnancy. A careful follow-up and guidance are necessary.

## 1 Introduction

Klippel–Trenaunay Syndrome (KTS) first described by Klippel and Trenaunay in 1900, which is a rare congenital disease characterized by cutaneous vascular malformations, venous varicosities, focal abnormalities of the deep venous system, and underlying soft tissue or bony hypertrophy^[1,2]^. It is a disease that increases obstetric risk and associates with exacerbation of these complications (thromboembolism and hemorrhagic) throughout pregnancy and postpartum. The present vascular alterations and the formations of new arteriovenous fistulas may be aggravated, consequent to postural and hormonal changes inherent to the pregnancy ^[3–7]^. Therefore, pregnancy is not advised in women with this syndrome. Being a rare disease, there are very few cases reported in peer-reviewed literature regarding pregnancy outcomes in women with KTS, moreover, it has never been reported in Asian woman.

Here, we report a case in an Asian pregnant patient with KTS, who had large varices in her pelvis, right lower limb, liver, and spleen, but had a successful vaginal delivery eventually without any complications at her 36^+6^ weeks of gestation.

## 2 Case presentation

A 31-year-old multipara (G3P1^+1^) presented at 28 weeks of gestation of her second pregnancy. She had extremity hemangioma on the right lower limb when she was only 3 months old and had no family history of KTS. She underwent a vascular embolization surgery at the age of 20 years. After that surgery, she gave a birth to baby by vaginal delivery at 26 years old without any complications during the gestation. No pelvic venous abnormality was found during her first pregnancy.

**Figure 1:**
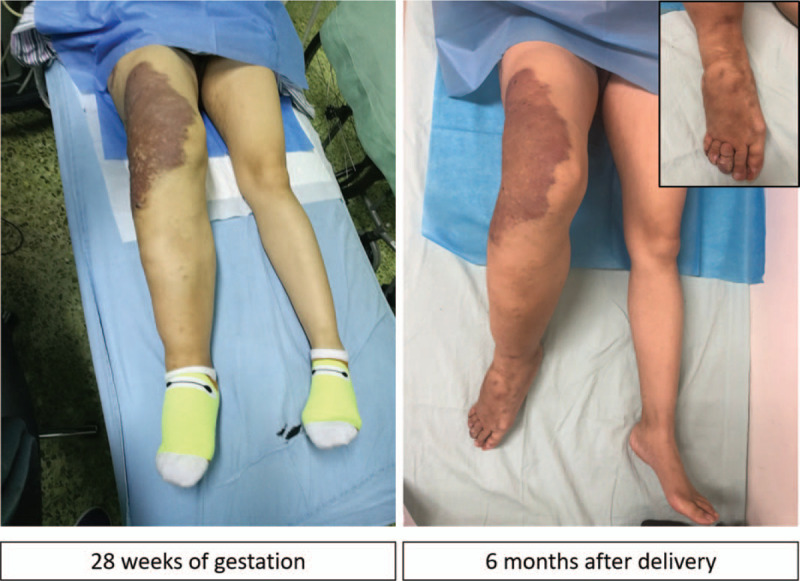
View of venous varicosities ad prominent hypertrophy on right lower limb during pregnancy and 6 months after delivery.

**Figure 2:**
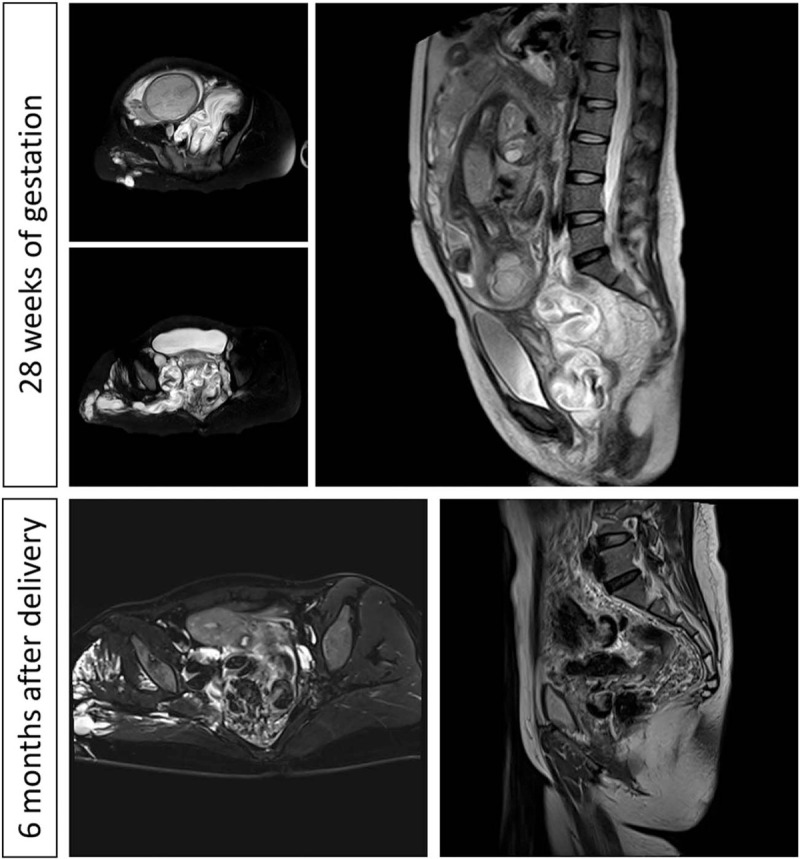
MRI views at 28 weeks of gestation and 6 months after delivery.

**Figure 3:**
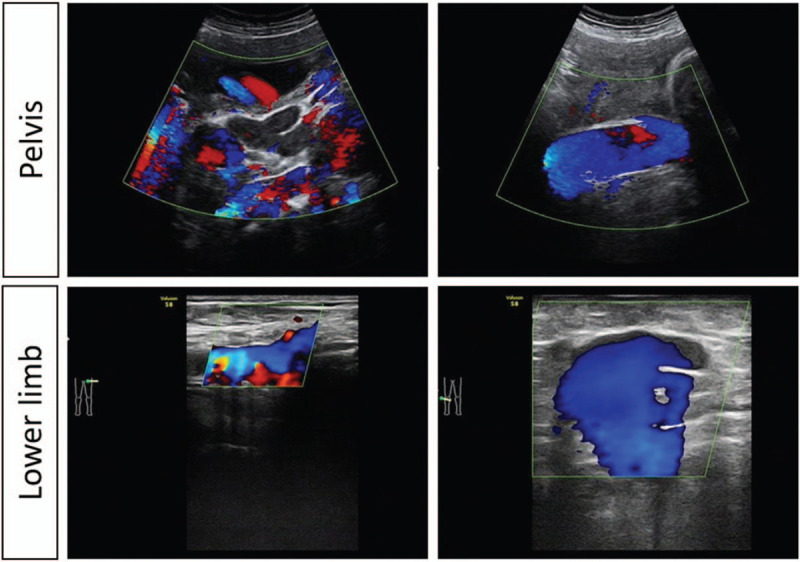
Ultrasonic view at 28 weeks of gestation.

During this pregnancy, she had a regularly obstetrics management at her first and second trimester in another local hospital before 28 weeks of gestation. Her pregnancy was uncomplicated until an ultrasound evaluation at 28 weeks of gestation at local hospital revealed a massive huge venous malformation behind cervix and in the pelvis. And she complained a gradually aggravated right sided limb pain. The local obstetrician transferred this patient to our hospital. During her hospital stay, she was systematically examined (Fig. 1). Physical examination showed prominent hypertrophy and multiple venous varicosities of the right leg. No obvious vulvovaginal varicosities were found. Laboratory studies revealed a normal coagulation profile (PT, 11.2 seconds; APTT, 39.1 seconds), and anemia (HGB, 65 g/L; Serum Ferritin, 9.2 ng/ml). Further gene examination found that she had α-Thalassemia and her husbands test was normal. A MRI of the abdomen, pelvis, and lower limb performed and revealed that a massive varicosity located behind cervix, and extended to the right lower limb (Fig. 2). No finding of vascularity was noted in spine or cranium area. The pelvic ultrasound examination revealed multiple massive (12.3 × 4.0 cm) vascular formation near to cervix in the pelvis (Fig. 3), as well as splenic and hepatic varicosities. On the basis of these findings, Blood transfusion and iron supplement were applied for the anemia correction. A multidisciplinary consulting was made with obstetrician, anesthesiologist and vascular surgeon for evaluating the risk of pregnancy and delivery. After counselling the risks of termination or continuous pregnancy and fully informed, the patient opted for continuous pregnancy. Vaginal delivery should be considered priority and general anesthesia should be suggested if a cesarean section was necessary with other obstetric indication. Low-molecular-weight heparin was administered. She was discharged after 11 days, and monitored with weekly obstetrics visit.

At 36^+6^ weeks of gestation, she presented to our hospital again with spontaneous pre-labor rupture of membranes, 1 cm cervical dilatation and 100% effacement. She was admitted to the labor room without any other obstetric indication for caesarean section. She delivered vaginally a female baby of 2920 g with Apgar score of 10, and 10 at 1, and 5 minutes with a minimal laceration. No abnormal finding indicative of neonatal KTS was observed. Even though a vaginal hemangioma (4 cm) was found in routine postnatal examination, her labor course was short and uneventful. The blood loss was about 350 ml, and no blood transfusion was required during this hospitalization. Both mother and baby were subsequently discharged 2 days after delivery. Her postnatal review at 6 weeks was uneventful with no complaints (Fig. 1). MRI and ultrasounds were evaluated again at 6 months after delivery. MRI revealed that the condition of the vascular anomalies in her pelvis and right lower limb showed no changes. No thrombus formation was observed by ultrasound examination as she had obvious enlarged right toes and increased pain in her right lower limb. A vascular surgery consult was arranged for the patient. But she rejected the vascular embolization surgery which the surgeon suggested strongly. This study has been approved by the Medical Ethics Committee of West China Second University Hospital of Sichuan University. Approval and informed consent from this patient have been obtained.

## 3 Discussion

Klippel–Trenaunay syndrome is a rare congenital disorder of unknown origin and variable expression affecting one or more extremities and characterized by extensive cutaneous hemangiomas and abnormal venous vessels. KTS in pregnant women is extremely rare, and very few cases have been published in literature ^[4–13]^. Physiological changes inherent to gestation, such as increase in circulating blood volume, weight, limb edema, changes in hormone levels, and venous obstruction by enlarged uterus worsen the capillary malformations and venous congestion present in the syndrome^[4]^. The etiology of KTS is not yet fully understood, and has regard to a few theories, which most likely the result of a somatic mosaicism: a postzygotic mutation that only affects a subset of the cells within body^[2]^. Increasing venous pressure, edema in lower extremities, venous stasis, and increasing cardiac output during pregnancy contribute to the increased risk of mainly thromboembolic and bleeding complications for the KTS patients.

We first reported pregnancy with KTS in Asian women, and our patient also differed from the previous cases, as varicosities were not only presented in the right lower limb, but also in liver, spleen, and pelvis. The massive vascularity behind cervix was as massive as 12.3 × 4.0 cm. In addition, she suffered from anemia with HGB only 65 g/L at her first hospitalization, which increased the risk of hemorrhage and made it difficult and risky to continue on pregnancy and delivery.

The mode of delivery needs to be individualized per case, and depends on multiple circumstances such as the previous history of the patient, including previous childbearing history, disorder related history which may indicate potential future complications and the imaging findings. The presence of varicosities in the cervical, vaginal, or vulvar area will make vaginal delivery difficult according to the high risk of rupture and hemorrhage. And anesthesia and vascular term consultation is strongly advised before delivery to optimize patient care and safety. Therefore, a multidisciplinary approach was necessary to decide the mode of delivery. There was no indication for cesarean section other than known obstetric indications. Thus, a vaginal delivery was recommended and a general anesthesia was considered if a cesarean section was necessary. In the present case, even though the vascular malformations were presented near to cervix, the course of labor was uneventful. Our patient was admitted to hospital due to pre-labor rupture of membranes at 36^+6^weeks, and delivered a healthy baby quickly. A minimal laceration was managed with a couple of sutures without any complications. A hemangioma as massive as 4 cm was noted in the vaginal postpartum, as there was no obvious vulvovaginal varicosities found during the hospitalization at 28 weeks gestation, which pointed out that hemangiomas could aggravate quickly during pregnancy. More frequent and careful observation should be managed to evaluate the KTS patients during pregnancy.

The main complication of KTS patients in pregnancy and postpartum is coagulopathy, including deep vein thrombosis and other thromboembolic problems^[4]^, which is 10 times greater risk than in the normal population^[2]^. Jacob et al reported that 8% if the affected patients develop pulmonary embolism or deep vein thrombosis^[14]^. There are no prospective studies investigating the use of the anticoagulant therapy during pregnancy in this rare disorder, but anticoagulant therapy, including low-dose aspirin, or low-molecular-weight heparin, is generally recommended to effectively prevent thromboembolic disease during pregnancy ^[10,15,16]^. Our patient was not followed up for many years after her first vascular embolization surgery 11 years ago and her first pregnancy 5 years ago. Further she rejected a vascular embolization surgery suggestion as her right lower toes enlarged obviously at her 6 months follow-up after delivery this pregnancy. Even though ultrasound revealed that no thrombus was found, it still reminded a very high risk of thrombus formation.

## 4 Conclusions

Pregnancy complicated by KTS is not an indication for termination of pregnancy. Our patient completed her second pregnancy without any complications and delivered a healthy baby. The success in the management of these patients with this syndrome requires the participation of a multidisciplinary team, consisting of obstetrician, anesthesiologist, urologist, and vascular surgeon. The use of prophylactic anticoagulants is generally advised during the pregnancy and postpartum period.

## Acknowledgments

The authors express their gratitude to the patient and her family for their support.

## Author contributions

**Conceptualization:** Bing Peng.

**Data curation:** Li Xiao, Bing Peng, Jinfeng Xu.

**Investigation:** Li Xiao, Bing Peng, Haibo Qu, Xiaohui Dai, Jinfeng Xu.

**Resources:** Haibo Qu, Xiaohui Dai.

**Writing – original draft:** Li Xiao.

**Writing – review & editing:** Bing Peng.
